# Hygienic Practices among Food Vendors in Educational Institutions in Ghana: The Case of Konongo

**DOI:** 10.3390/foods2030282

**Published:** 2013-07-09

**Authors:** Isaac Monney, Dominic Agyei, Wellington Owusu

**Affiliations:** 1 Department of Environmental Health and Sanitation, University of Education, Winneba, P.O. Box M40, Mampong Ashanti, Ghana; E-Mail: wellington.owusu@yahoo.com; 2 Bio Engineering Laboratory, Department of Chemical Engineering, Monash University, Melbourne, 3800, Australia; E-Mail: Dominic.Agyei@monash.edu

**Keywords:** food hygiene, food safety, vendors, sanitary conditions, Konongo, Ghana

## Abstract

With the booming street food industry in the developing world there is an urgent need to ensure food vendors adhere to hygienic practices to protect public health. This study assessed the adherence to food hygiene practices by food vendors in educational institutions in Konongo, Ghana. Structured questionnaires, extensive observation and interviews were used for the study involving 60 food vendors from 20 basic schools. Attributable to the influence of school authorities and the level of in-training of food vendors, the study points out that food vendors in educational institutions generally adhered to good food hygiene practices, namely, regular medical examination (93%), protection of food from flies and dust (55%); proper serving of food (100%); good hand hygiene (63%); and the use of personal protective clothing (52%). The training of food vendors on food hygiene, instead of the level of education had a significant association (*p* < 0.05) with crucial food hygiene practices such as medical examination, hand hygiene and protection of food from flies and dust. Further, regulatory bodies legally mandated to efficiently monitor the activities of food vendors lacked the adequate capacity to do so. The study proposes that efforts should be geared towards developing training programmes for food vendors as well as capacity building of the stakeholders.

## 1. Introduction

Street foods are very well patronized in many developing countries since they are affordable, easily accessible and also serve as an important source of income [1,2]. However, these street foods largely do not meet proper hygienic standards and can therefore lead to morbidity and mortality due to food borne illnesses, and concomitant effects on trade and development [1,3].

Food-borne illnesses are a growing public health concern worldwide and results from food contaminated by pathogenic microorganisms, mycotoxins or chemical hazards [3]. This concern is heightened by the fact that, worldwide, there seems to be a change in life-style and food consumption patterns as frequency of “eating out” is increasing and commitment to food preparation at home is decreasing [4].

The number of reported outbreaks of food-borne illnesses has been high, both in developed as well as developing countries [4,5]. However, the problem is exacerbated in developing countries due to economic reasons, poverty, the lack of adequate health care facilities, and the dearth of data regarding food-borne diseases. This greatly compromises the achievement of the Millennium Development Goals (particularly MDG 1, 4, 5 and 6) [3]. The safety of street or vended foods is therefore one of the most pressing health and safety issues facing most developing countries since it leads to both public health and social consequences.

Food contamination in developing countries is caused by many factors including traditional food processing methods, inappropriate holding temperatures, and poor personal hygiene of food handlers [2]. Further, the prevalence of food-borne illnesses in developing countries is intertwined with other economic and developmental issues, namely, legislation, infrastructure and enforcement mechanisms. Specific examples include inadequacy of food safety laws, laxity in regulatory enforcements, and the lack of education for food handlers [1]. The incidence of food- and water-borne diseases is estimated at 3.3–4.1 episodes per child per year in Africa and food and water-borne diarrhoeal diseases are estimated to cause between 450,000–700,000 deaths in Africa annually, with many more sporadic cases going unrecorded [6,7]. In most of these cases, pathogens such as *Escherichia coli*, *Bacillus cereus*, *Salmonella*, Hepatitis, *Shigella*, *Brucella*, *Staphylococcus aureus*, *Campylobacter*, rotavirus and enteric bacteria are identified [1,2].

In Ghana, as well as in many countries in the African region, there is an abundance of national legislation but limited resources to control street food safety [1]. Institutions such as the Ghana Standards Authority and the Food and Drugs Board are committed to the work of regulating food standards and training the general populace on food safety issues; however, improvement in food safety systems has not been fully realized and this is observed in recent reports of food borne illness and/or contamination of street foods with enteric bacteria in various parts of the country [2,8].

A number of outbreaks have recently been reported in Ghana. For example, four persons died in Sheho (Upper East Region of Ghana) after eating contaminated meat [9]. Also, a cholera outbreak in Atebubu (Brong Ahafo Region) claimed nine lives [10] while another such outbreak resulted in the death of one person in Obuasi (Ashanti Region) and the hospitalization of over 50 [11]. It has been estimated that about 5000 children under five years of age die from diarrhoea each year in Ghana [12]. Despite these alarming statistics, only few surveys have been done to understand and correlate the causes of food-borne illnesses in Ghana.

Food vendors may contaminate food by poor personal hygiene, cross-contaminating raw and processed food, as well as inadequate cooking and improper storage of food [4]. Maintaining high food safety levels in school food services is very important because any incidences can affect a high number of students [5]. The objective of this study therefore was to evaluate the hygienic practices and sanitary conditions of food vendors in some basic schools in a municipal capital town in Ghana.

## 2. Methodology

**Figure 1:**
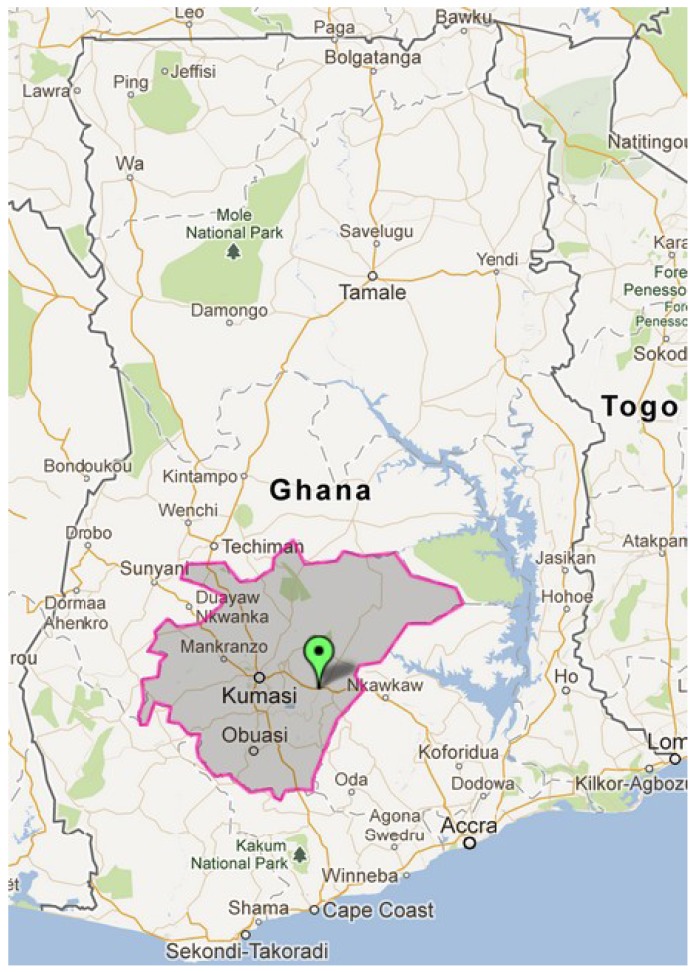
Location of Konongo in the Ashanti region of Ghana (N 8.58102 W 0.68115); Courtesy: Zee maps [13].

Konongo is located in the Ashanti Region of Ghana and serves as the capital of the Asante Akim Central Municipality. Geographically, it lies on latitude 6°38′29″N and longitude 1°16′03″W and has a population of approximately 25,000. Konongo is about 53 km from Kumasi, the Ashanti regional capital and 200 km from Accra, the national capital. Konongo is endowed with rich gold deposits and thus, mining is an important activity in the town. As a result immigration of human labour, particularly the youth, is a common feature in Konongo. Figure 1 shows the location of Konongo in the Ashanti Region of Ghana (highlighted).

The study drew predominantly on a descriptive survey using structured questionnaires and extensive observation of vendors to assess the hygienic practices of food vendors in educational institutions and the sanitary conditions at the food vending points. Moreover, through interviews with school authorities and the Environmental Health Department, it examined the role of these core stakeholders in ensuring that food vendors conduct their activities in a safe and hygienic manner. Specifically, the study parameters assessed included the level of education, the means of acquiring skills, the personal hygiene of food vendors (hands, nails, and hair), the use of protective garments, the means of food protection, and the availability of medical certificates. In all, 60 food vendors in 20 schools, school authorities and the Chief Environmental Health Officer of the Asante Akim Central Municipality were involved in the study. All 60 food vendors involved in the study were stationary food vendors selling ready-to-eat food in educational institutions in Konongo. Only food vendors who prepared food at home and conveyed them to the school premises to be sold were used for the study. Those who sold pre-packaged foods were excluded in this study.

The study questionnaire was organized into distinctive sections to obtain information pertaining to respondents’ socio-demographic characteristics; level of knowledge and training on food hygiene; status of medical screening and an observation checklist to determine personal hygiene and food handling practices. The heads of all twenty educational institutions involved in the study were also interviewed. It was categorized into two major aspects, namely, the roles of the authorities in ensuring adherence to food hygiene practices among food vendors; and the disciplinary actions taken against food vendors who flout their directives on food vending. The results from the study (quantitative data) were analysed with the Statistical Package for Social Sciences (SPSS, Version 16.0, [14]) using descriptive statistics such as frequencies and percentages. Comparative analysis to determine associations between study parameters were carried out with the chi square (*χ*^2^) test at 5% significance level.

## 3. Results and Discussion

The results of the study are given as follows. All food vendors surveyed were females and this agrees with findings from Lues *et al.* [15] who found street food vending to be a common income-generating venture particularly for women in developing countries. Half of the food vendors30 (50.0%) were 25–35 years of age while 2 (3.3%) were in the age group of 45–55 years as shown in Table 1. Food vendors below 18 years were not found in the educational institutions since the school authorities regard it as a form of child labour. This confirms a study by Musa and Akande [16] who found a low level of involvement of under-aged food vendors in educational schools in Ilorin, Nigeria.

Respondents had at least primary school education (5%), with almost half of them (48.0%) attaining senior high school (SHS) education and (37.0%) attaining junior high school (JHS) education. A good proportion of respondents 31 (51.7%) had been selling food for less than five years and the proportion of respondents reduces with increasing number of years (Table 1); meaning, in the Konongo study area, the street food business has boomed only recently. This trend is similar to that reported by Abdalla *et al.* [17] and confirms the assertions in available literature [16,18] that the food vending business in developing countries is rapidly expanding and serves as a form of employment for urban residents.

**Table 1:** Characteristics of respondents.

Study parameter		Frequency (*n* = 60)	Percentage (%)
Gender	Females Males	60 0	100.0 0.0
Age group (years)	18–25 25–35 35–45 45–55	3 30 25 2	5.0 50.0 41.7 3.3
Level of education	None Primary Junior high school Senior high school Vocational school	0 3 22 29 6	0.0 5.0 37.0 48.0 10.0
Period of selling food	Less than 5 years 5–10 years 10–15 years More than 15 years	31 20 5 4	51.7 33.3 8.3 6.7

Approximately 86.7% of the vendors learned their trade through their own personal intuition and informal education from friends and parents whilst 13.3% acquired their skills formally from vocational institutions and senior high school (Table 2). This disagrees with studies in Nigeria by Chukuezi [19] where only 5% of street food vendors (*n* = 63) had formal education. The majority of food vendors (65%) asserted that they had received on-the-job training on food hygiene by the Food and Drugs Authority and the Municipal Assembly whiles 35% had not received any training on food hygiene, as detailed in Table 2. According to FAO and WHO [20], food vendors are required to undergo basic training in food hygiene before licensing and further training as required by the relevant authority. This is because inadequate hygiene training and/or instruction and supervision of all people involved in food related activities poses a potential threat to the safety of food and its suitability for consumption [21]. Considering the mode of acquisition of skills for the sale of food for most of the vendors (86.7%), the need for further training on food hygiene is extremely crucial due to the fact that they may not have adequate knowledge on hygienic practices with regard to their trade.

**Table 2:** Respondents’ level of knowledge on food hygiene.

Study parameter		Frequency (*n* = 60)	Percentage (%)
Acquisition of knowledge on food preparation and vending	Informal education/self-taught Formal education/vocational institution	52 8	86.7 13.3
Training on food hygiene and safety (formal)	Yes No	39 21	65.0 35.0
Informal education on food hygiene and safety by school authorities	Yes No	52 8	87.0 13.0
Awareness of laws on food hygiene and safety	Yes No	46 14	77.0 23.0

Regarding the level of knowledge on food safety 77% of the vendors had some knowledge on laws regarding food hygiene, while 23% had no knowledge thereof. In Ghana, the Food and Drugs Law (PNDC Law 305 B) [22], Amendment Act 523 [23] and various bye-laws on food hygiene aim at ensuring that only safe and wholesome food, drugs and other substances are made available for public consumption. As per these laws, the sale of food under unsanitary conditions is an offence. All food vendors with some knowledge of laws regarding food sale (77%) were aware of this aspect of the law. It was also observed that the schools provided education on food hygiene and safety for food vendors, wherein 87% of food vendors asserted themselves to have been educated by school authorities on food hygiene (Table 2).

Medical examination of food handlers, as per FAO and WHO [21], is necessary if clinically or epidemiologically indicated. This is to ensure that people with communicable diseases are excluded from food handling. Conversely, Abdulssalam and Kaferstein [24] argue that medical examination of food vendors prior to licensing, or at intervals afterwards, does little towards ensuring food safety and should not be mandatory. All the same, as a form of precaution, Section 286 of the Criminal Code, (Amendment) Act, 2003 (Act 646) of Ghana charges all food vendors to be examined to ensure they do not infect consumers with communicable diseases [25].

Results from the study show that 68% of the vendors had been medically examined, out of which 95% showed their certificates as evidence during the study while the remaining 5% could not readily produce their certificate at the time of the interview (Table 3). Ninety-three per cent (93%) and 7% of the vendors performed their medical examination in the years 2012 and 2011 respectively. Among the vendors who had been medically examined, 34.0% complained of difficulties owing to factors such as delay in the laboratory results, poor services rendered at the laboratory and high cost of laboratory services (Table 3). According to the food vendors, these are among the factors that discourage vendors from availing themselves for medical screening.

**Table 3:** Information on medical screening of respondents.

Study parameter		Frequency	Percentage (%)
Medical examination (*n* = 60)	Yes No	41 19	68.0 32.0
Most recent date of examination (*n* = 41)	2011 2012	3 38	7.0 93.0
Evidence of medical examination (*n* = 41)	Yes No	39 2	95.0 5.0
Difficulty in acquiring medical examination certificate (*n* = 41)	Yes No	14 27	34.0 66.0
Nature of difficulty encountered (*n* = 14)	Delay in results Expensive Poor laboratory services	12 1 1	86.0 7.0 7.0

Approximately, 52.6% of food vendors without medical examination certificates (*n* = 19) stated that they were not aware of the requirement whereas others gave reasons such as lack of finances (15.8%), unnecessary (15.8%) and too busy to make time for medical screening (15.8%) as justification for not having been medically examined.

**Figure 2:**
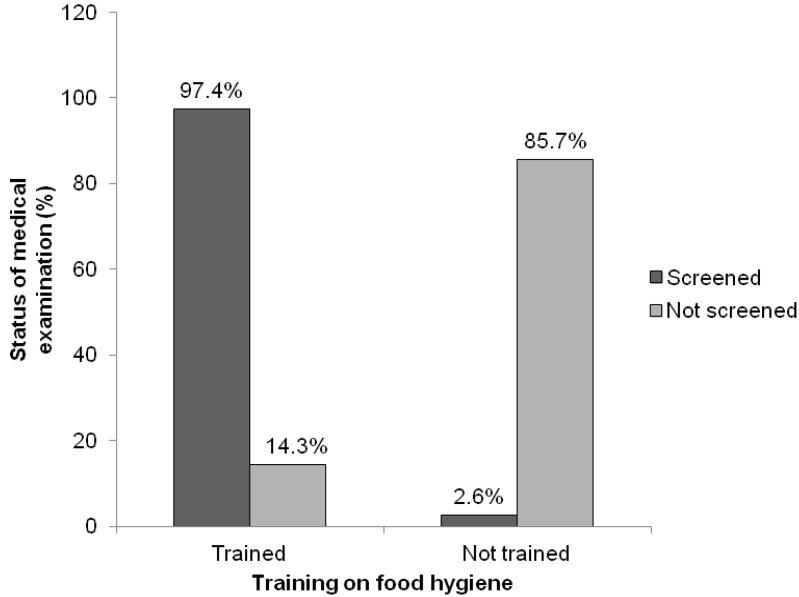
Comparative analysis between training on food hygiene and status of medical examination.

Previous training on food hygiene influenced the likelihood of medical examination among food vendors. It was revealed that a good proportion (97.4%) of food vendors who had been trained on food hygiene, had been medically screened, whereas only 2.6% of those without any training on food hygiene had been medically examined (Figure 2). This was found to be statistically significant (*p* < 0.05) from the chi square test.

About 55.0% of vendors were observed to adequately protect their food from flies and dust whilst 45.0% had no protection, thus exposing their food to flies and dust (Table 4). This is somehow disconcerting given that the FAO and WHO [20] recommend that food should be adequately protected from airborne contaminants and pests in such a way as not to pose a threat to food safety.

**Table 4:** Hygienic practices observed by food vendors.

Observed conditions		Frequency (*n* = 60)	Percentage (%)
Adequate protection of food from flies and dust	Yes	33	55.0
	No	27	45.0
Dishing out food	Spoon/ladle	60	100.0
	Bare hand	0	0.0
Presence of food debris on vendors’ hands	Yes	39	65.0
	No	21	35.0
Finger nails	Clean	38	63.3
	Unclean	22	36.7
Hair protection	Present	9	15.0
	Absent	51	85.0
Use of apron	Yes	31	52.0
	No	29	48.0

**Figure 3:**
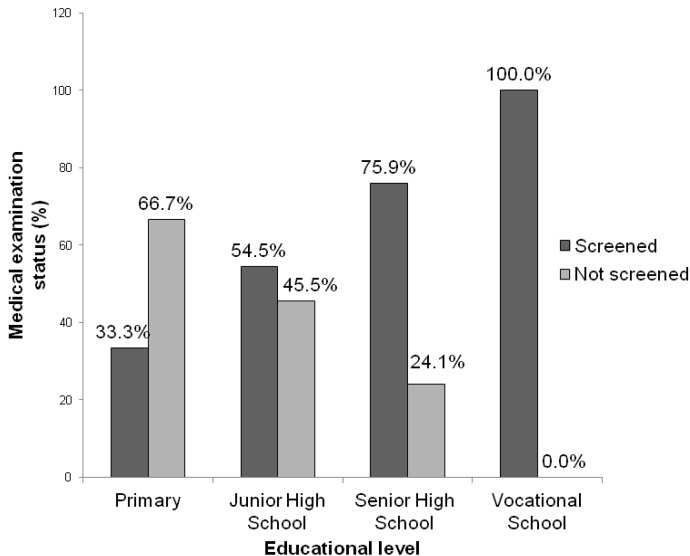
Relationship between vendors’ educational level and status of medical examination.

Results of the chi-square test carried out to establish the relationship between the level of education and the status of medical examination revealed no statistical difference (*p* = 0.067). This indicates that the educational level of food vendors and the inclination to go for medical examination are not necessarily correlated. However, formal education is important for food vendors. Figure 3 shows that, 33.0% of food vendors with primary school education had been medically screened whereas all those with vocational training had been medically screened thereby underscoring the importance of formal education prior to food vending.

None of the vendors involved in the study were seen to dish out food with bare hands but rather used either a spoon or ladle. This differs from findings by Muinde and Kuria [26] in Nairobi who reported that 60% of street food vendors (*n* = 80) handled food with their bare hands. Conversely, it was observed that 65% of the vendors had food remains on their hands, indicating possible hand contact with food during dishing out with spoon or ladle. According to Ferron *et al.* [27], the hands of food vendors are usually the most critical means of transmitting pathogens from contaminated places and items and hence could result in cross contamination upon contact with food. Particularly, in the case where vendors use the same hands to handle money from consumers, as this can further aggravate the situation due to possible accumulation of dirt on the money.

In addition, 63% had clean, short and well trimmed fingernails, 15% had hair restraints in the form of a head scarf and 52% wore aprons (Table 4). Rane [28] reported that *Salmonella*, non-typhi salmonellae, *Campylobacter* and *E. coli* can survive on finger tips and other surfaces for different periods of time and in some instances even after hand washing. It therefore behooves on food vendors always to keep their finger nails short and clean to prevent them from serving as a vehicle for transmission of pathogens [16]. The low proportion of food vendors with hair restraints, as found in this study, is in contrast with the findings of Musa and Akande [16] but in corroboration with those reported by Muinde and Kuria [26] and Abdalla *et al.* [17] who reported a relatively low level of hair protection by food vendors. The World Health Organization (WHO) has however asserted that as a practice, the use of aprons and hair restraints by food vendors has more to do with food aesthetics and stimulating consumer assurance than food safety [29].

All food hygiene variables were not associated with the level of education as shown in Table 5. This confirms findings by Mwangi [30] who reported no statistically significant differences between the level of education and the hygienic practices of food vendors. Thus, the level of education of food vendors does not necessarily affect their inclination to observe food hygiene practices such as protection of food from flies and dust, hair protection, cleanliness of fingernails and use of an apron.

**Table 5:** Relationship between vendors’ educational level and food hygiene variables.

Food hygiene variables		Highest level of education			
		Primary school	Junior high school	Senior high school	Vocational training
Protection of food from flies and dust (*p* = 0.434)	Yes	33.3%	50.0%	55.2%	83.3%
	No	66.7%	50.0%	44.8%	16.7%
Presence of food debris on vendors’ hands (*p* = 0.287)	Yes	66.7%	63.6%	58.6%	100.0%
	No	33.3%	36.4%	41.4%	0.0%
Finger nails (*p* = 0.741)	Clean	33.3%	63.6%	65.5%	66.7%
	Unclean	66.7%	36.4%	34.5%	33.3%
Hair protection (*p* = 0.419)	Present	0.0%	9.1%	17.2%	33.3%
	Absent	100%	90.9%	82.8%	66.7%
Use of apron (*p* = 0.809)	Present	33.3%	50.0%	51.7%	66.7%
	Absent	66.7%	50.0%	48.3%	33.3%

A comparative analysis from the chi-square test indicates that the cleanliness of vendors’ finger nails as well as the adequacy of food protection from flies is associated with training of food vendors on food hygiene and safety since it showed a statistically significant difference (Table 6). This shows that food vendors trained on food hygiene and safety are more likely to keep their finger nails clean and adequately protect their food from flies and dust.

**Table 6:** Relationship between training on food hygiene and safety, cleanliness of fingernails and extent of food protection among food vendors.

		**Condition of finger nails, *n* (%)**		
		**Clean**	**Unclean**	**Total**
Training on food hygiene and safety (*p* = 0.016)	Trained	29 (74.4%)	10 (25.6%)	39
	Not trained	9 (42.9%)	12 (57.1%)	21
		**Protection of food from flies and dust, *n* (%)**		
		**Yes**	**No**	**Total**
Training on food hygiene and safety (*p* = 0.001)	Trained	28 (71.8%)	11 (28.2%)	39
	Not trained	5 (23.8%)	16 (76.2%)	21

Training of food vendors on food hygiene and safety did not have a statistically significant association (*p* >0.05) with hair protection, use of an apron and accumulation of food debris on vendors’ hands (Table 7). However, there was a relatively higher tendency for vendors trained on food hygiene and safety to prevent hand contact with food during dishing out.

**Table 7:** Relationship between training on food hygiene and safety, hair protection, use of apron and accumulation of food debris on vendors’ hands.

		**Hair covering, *n* (%)**		
		**Yes**	**No**	**Total**
Training on food hygiene and safety (*p* = 0.909)	Trained	6 (15.4%)	33 (84.6%)	39
	Not trained	3 (14.3%)	18 (85.7%)	21
		**Use of apron, *n* (%)**		
		**Yes**	**No**	**Total**
Training on food hygiene and safety (*p* = 0.316)	Trained	22 (56.4%)	17 (43.6%)	39
	Not trained	9 (42.9%)	12 (57.1%)	21
		**Presence of food debris on vendors’ hands; *n* (%)**		
		**Yes**	**No**	**Total**
Training on food hygiene and safety (*p* = 0.182)	Trained	23 (59%)	16 (41%)	39
	Not trained	16 (76.2%)	5 (23.8%)	21

Findings from interviews of authorities in the twenty educational institutions involved in the study point out that, as part of their efforts to promote food safety, they ensure that all food vendors within their premises are medically certified before being allowed to vend food in their schools. The authorities also asserted that they occasionally inspect food vending premises and educate food vendors on basic food hygiene and safety principles. Among their informal regulations for food vendors, those found selling under unhygienic conditions and without the appropriate medical certification are initially cautioned and recalcitrant vendors are prohibited from food vending within their premises.

This, perhaps, explains the relatively higher levels of food hygiene practices among food vendors in the educational institutions as opposed to the poor food hygiene practices among those found on the streets as reported in studies by various authors [2,26,28,30]. However, it became apparent in this study that no food vendor had ever been stopped on these grounds. Further enquiries into this phenomenon revealed that, in most cases, food vendors are related to an employee of the school making it difficult for such action to be taken against her.

The Environmental Health and Sanitation Departments in all districts in Ghana are legally mandated by the Food and Drugs Law (PNDC Law 305 B) [22], Amendment Act 523 [23]; Section 286 of the Criminal Code, 1960 (Act 29) of the Republic of Ghana [25]; Local Government Instrument, 1995-LI 1615 [31] and other District bye-laws to among others, conduct regular inspection of food vending premises and prosecute vendors who sell food under unhygienic conditions [22,32]. In spite of these mandates, it was pointed out through an interview with the Chief Environmental Health Officer of the Asante Akim Central Municipality that the department lacks the capacity (human resource, transportation and funds) to efficiently monitor the activities of food vendors within its jurisdiction. This, together with other factors, has constrained their efforts to effectively regulate, monitor and supervise the activities of street-food vendors and ensure public health protection.

## 4. Conclusions

The study found that food vendors in educational institutions generally adhered to good food hygiene practices with regards to regular medical examination, protection of food from flies and dust, serving of food, hand hygiene and use of an apron. The study pointed out that there is no significant association (*p* > 0.05) between the vendors’ level of education and their acceptance to undergo medical examination and no statistically significant relationship was found between the education level and food hygiene practices. Training of food vendors on food hygiene and safety had a significant association with crucial food hygiene and safety practices such as medical examination, hand hygiene and protection of food from flies and dust. This underscores the importance of training among food vendors to ensure perpetuation of best practices in the street food vending business thereby protecting public health. Development of training programmes for food vendors is therefore highly recommended. Ideally, this should be carried out at no cost to food vendors and a certificate should be awarded at the end of each training programme. Training manuals should be developed for trainers to serve as a guide and ensure uniformity of subject matter. Impact analysis of training programmes in achieving and sustaining behavioural change among food vendors should also be carried out through regular monitoring. This can be done by adequately resourcing the Environmental Health and Sanitation Departments country-wide with funds, human resources and logistics to enhance their monitoring and evaluation activities. Authorities in educational institutions in Konongo, Ghana should be made aware of the vital role they play in ensuring adherence to food hygiene practices among food vendors on their premises. Formation of local food vendor groups would also ensure that food vendors adhere to appropriate codes of practice in street food vending and also serve as a vehicle to efficiently train and convey information to food vendors. 

## References

[1] DeWaal C.S., Rober N. Global and Local: Food Safety around the World. http://safefoodinternational.org/local_global.pdf.

[2] Feglo P., Sakyi K. (2012). Bacterial contamination of street vending food in Kumasi, Ghana. J. Med. Biomed. Sci..

[3] Food and Agriculture Organization of the United Nations, World Health Organization Assuring Food Safety and Quality: Guidelines for Strengthening National Food Control Systems. http://www.who.int/foodsafety/publications/capacity/en/Englsih_Guidelines_Food_control.pdf.

[4] Initiative to Estimate the Global Burden of Foodborne Diseases. http://www.who.int/foodsafety/foodborne_disease/ferg/en/index.html.

[5] Osaili T.M., Abu Jamous D.O., Obeidat B.A., Bawadi H.A., Tayyem R.F., Subih H.S. (2013). Food safety knowledge among food workers in restaurants in Jordan. Food Control.

[6] Santos M.-J., Nogueira J.R., Patarata L., Mayan O. (2008). Knowledge levels of food handlers in portuguese school canteens and their self-reported behaviour towards food safety. Int. J. Environ. Health Res..

[7] Simpson E., Wittet S., Bonilla J., Gamazina K., Cooley L., Winkler J. (2007). Use of formative research in developing a knowledge translation approach to rotavirus vaccine introduction in developing countries. BMC Public Health.

[8] Todd E.C. (1997). Epidemiology of foodborne diseases: A worldwide review. World Health Stat. Q..

[9] Ghana News Agency Contaminated Food, Water Causes 700,000 Deaths in Africa Annually. http://www.modernghana.com/news/203772/1/contaminated-food-water-causes-700000-deaths-in-af.html.

[10] GhanaWeb. Four Dead after Eating Contaminated Food. http://www.ghanaweb.com/GhanaHomePage/NewsArchive/artikel.php?ID=195505#.

[11] JoyNews. Nine Confirmed Dead in Cholera Outbreak at Atebubu. http://edition.myjoyonline.com/pages/news/201207/89610.php.

[12] GraphicOnline. Obuasi Battles Cholera Outbreak. One Dead So Far. http://graphic.com.gh/Health/obuasi-battles-cholera-outbreakone-dead-so-far.html.

[13] ZeeMaps. http://www.zeemaps.com/.

[14] SPSS Version 16. http://www-01.ibm.com/software/analytics/spss/.

[15] GraphicOnline. Global Study Suggests Solutions to Childhood Diarrhoea. http://graphic.com.gh/Health/global-study-suggests-solutions-to-childhood-diarrhoea.html.

[16] Lues J.F.R., Rasephei M.R., Venter P., Theron M.M. (2006). Assessing food safety and associated food handling practices in street food vending. Int. J. Environ. Health Res..

[17] Musa O.I., Akande T.M. (2003). Food hygiene practices of food vendors in secondary schools in Ilorin. Niger. Postgrad. Med. J..

[18] Abdalla M.A., Siham E.S., Alian Y.Y.H.A., Amel O.B. (2008). Food safety knowledge and practices of street food-vendors in Khartoum city. Sudan J. Vet. Sci. Anim. Husb..

[19] Rheinländer T., Olsen M., Bakang J., Takyi H., Konradsen F., Samuelsen H. (2008). Keeping up appearances: Perceptions of street food safety in urban Kumasi, Ghana. J. Urban Health.

[20] Chukuezi C.O. (2010). Food safety and hyienic practices of street food vendors in Owerri, Nigeria. Stud. Sociol. Sci..

[21] (1997). General Requirements (Food Hygiene). Joint FAO/WHO Food Standards Programme, Codex Alimentarius Commission, Rome, Italy. http://www.fao.org/docrep/w6419e/w6419e00.HTM#Contents.

[22] (1992). Food and Drugs Act (PNDC Law 305 B); Parliament of the Republic of Ghana, Accra, Ghana. http://www.epa.gov.gh/ghanalex/acts/Acts/FOOD%20AND%20DRUGS%20BOARD.pdf.

[23] (1996). Food and Drugs (Amendment) Act 523, Parliament of the Republic of Ghana, Accra, Ghana. faolex.fao.org/docs/pdf/gha17283.pdf.

[24] Food and Agriculture Organization of the United Nations, World Health Organization (2009). Food Hygiene Basic Texts.

[25] Section 286. The Criminal Code of Ghana (Amendment) Act, 2003 (Act 646). http://www.refworld.org/docid/44bf823a4.html.

[26] Abdussalam M., Kaferstein F.K. (1993). Safety of street foods. World Health Forum.

[27] Muinde O.K., Kuria E. (2005). Hygiene and sanitary practices of vendors of street foods in Nairobi, Kenya. Afr. J. Food Agric. Nutr. Dev..

[28] Ferron S., Morgan J., O’Reilly M. (2000). Hygiene Promotion: A Practical Manual for Relief and Development.

[29] Rane S. (2011). Street vended food in developing world: Hazard analyses. Indian J. Microbiol..

[30] World Health Organization (1996). Essential Safety Requirements for Street-Vended Foods.

[31] (1995). Local Government (Accra Metropolitan Assembly Establishment) Instrument, 1995-LI 1615.

[32] Mwangi A.M. (2002). Nutritional, Hygienic and Socio-Economic Dimensions of Street Foods in Urban Areas: The case of Nairobi.

